# Erythema nodosum: what should we consider about it?

**Published:** 2016

**Authors:** Farhang Babamahmoudi, Arghavan Amuzgar, Tahoora Mousavi, Lotfollah Davoodi

**Affiliations:** 1 Department of Infectious Diseases, University of Medical Sciences, Sari, Iran.; 2 Molecular and Cell Biology Research Center, Student Research Committee, Faculty of Medicine, Mazandaran University of Medical Sciences, Sari, Iran

**Keywords:** Erythema nodosum, Panniculitis, Erythematous

**Dear Editor,**

Erythema nodosum (EN) or panniculitis is a cutaneous reaction consisting of inflammatory tender erythematous nodular lesions, spontaneously regressing located primarily over the extensor surface of lower extremities (1). EN can occur at any age but its peak incidence is between the third and fourth decades (2). Prevalence of EN is 3-5 times higher in women than men (3). Erythema nodosum is panniculitis and associated with variety of conditions such as: infectious disease(4), drugs (5), pregnancy (6), autoimmune disease (7), idiopathic (8). 

This study was carried out at the Central Infectious unit of a university hospital (Quaemshahr Razi) between 2012 to 2015. In 53 probable cases, 21 patients were confirmed cases with biopsy samples, and the others were excluded from the study. Their ages was above 14 years old comprising 5 males and 16 females (sex ratio 3.2/1). The evaluation began by taking physical examination, family history and drug history and underlying conditions. The patient underwent a complete laboratory.

In our finding, all patients, skin biopsy sample that showed panniculitis were taken. Table 1 shows the probable underlying disease. In this study, in one female patient, all her four extremities had EN while in 2 females, their three extremities (bilateral upper extremities with on lower limb) were affected. In patients, the rise of LFT tests (liver function tests AST and ALT) and rise of renal function tests (blood urea and creatinin) were not detected.

In a prospective study that was carried out at the University Hospital of Ioannina, almost all (98%) patient had EN over the extensor surface of lower extremities, mainly on the shins and bilaterally in 82%. Our finding was different as described above (6). In one finding in Strasburg, data confirmed the predominance of streptococcal infections and sarcoidosis among patients with EN. Tuberculosis virtually disappeared, since the last case was observed in 1962. Various viral or bacterial disease are performed to determine the true prevalence of EN related diseases (9). Another study in Verona, Italy showed that in 58.8% patients, an etiology of the first manifestation of EN was attributed to infections (25.8% of total number; 32% of those with an attributed etiology), drugs(mostly sex hormones; 15.35%; 26%), systemic disease (11.2%;19.2%) and pregnancy (6.5%; 10.9%). EN relapsed in 33 (26.6%) patients and was mostly attributed to infections and drugs. Factors responsible for the first manifestation of EN frequently differed from those causing relapses in the same patients, with the exception of drug-induced EN. In this study, we conclude that drug-induced EN can recur after re-exposure to the same drug, and the recurrence can be predicted (10).

**Table 1:** The probable underlying disease

**Etiology **	**Female 16(76%),** **16-76 years**	**Male 5(24%),** **12-76 years**	**Total**
Brucellosis	3(14%)	1(20%)	4(19.4%)
Granulomatos mastitis	2(12.5%)	0(0%)	2(9.52%)
Oral contraceptives	3(14%)	0(0%)	3(14.28%)
EN in pregnancy	2(12.5%)	0(0%)	2(9.52%)
Sarcoidosis	0(0%)	1(20%)	1(4.76%)
IBD(ulcerative colitis)	1(6.25%)	0(0%)	1(4.76%)
Atypical pneumonia	2(12.5%)	0(0%)	2(9.52%)
Cellulitis	0(0%)	1(20%)	1(4.76%)
Streptococcal pharyngitis	1(6.25%)	0(0%)	1(4.76%)
Idiopathic	2 (12.5%)	2(40%)	4(19.4%)
Bilateral leg EN	7(43.75%)	4(80%)	11(52%)
Unilateral leg EN	5(31.25%)	2(40%)	7(33%)
Increase ESR	13(81.25%)	3(60%)	16(76%)
Increase CRP	8(50%)	4(80%)	12(57%)
Increase ESR and CRP	6(37.5)	2(40%)	8(38%)
Increase wright , 2ME , coombs wright	3(14%)	1(20%)	4(19.4%)
Increase WBC count	2(12.5%)	1(20%)	3(14%)
Atypical pneumonia	2(12.5%)	0(0%)	2(9.52%)
Rise of ASO (anti streptolyzin)	1(6.25%)	0(0%)	1(4.76%)
Increase ACE level (angiotensin converting enzyme)	0(0%)	1(20%)	1(4.76%)
Sum	16	5	21

In this investigation, the underlying etiology that we found was different from other studies, probably due to epidemiologic differences in each region. Thus, we suggest physicians consider endemic disease, such as brucellosis in patients with E.N to avoid misdiagnosing. General lab data consisting CBC, electrolities, LFT, etc. did not have diagnostic role in our study and each patient was only diagnosed when we tested a specific etiology (Wright, ACE, ASO, etc). Although, we should not forget that the first key is the complete history taking and physical examination.
